# Bioaccumulation of Cr by the *Buddleja* Species and *Schinus molle* L. Grown with and Without Compost in a Sandy Soil Contaminated by Leather Industrial Effluents

**DOI:** 10.3390/plants13243469

**Published:** 2024-12-11

**Authors:** Jamilet Huarsaya-Huillca, Sheyla Callo-Sánchez, Camila Aguilar-Ccuno, Oswaldo Rodríguez-Salazar, Danny Tupayachy-Quispe, Giuliana Romero-Mariscal, Zulema Hachire-Patiño, Jonathan Almirón

**Affiliations:** 1 Escuela Profesional de Ingeniería Ambiental, Universidad Nacional de San Agustín de Arequipa, Calle Santa Catalina N°117 Cercado, Arequipa 04001, Peru; jhuarsayah@unsa.edu.pe (J.H.-H.); scallos@unsa.edu.pe (S.C.-S.); caguilarcc@unsa.edu.pe (C.A.-C.); gromeroma@unsa.edu.pe (G.R.-M.); 2 Laboratorio de Ciencia de los Materiales, Facultad de Ciencias e Ingenierías Físicas y Formales, Universidad Católica de Santa María, Samuel Velarde 320, Arequipa 04000, Peru; orodriguez@ucsm.edu.pe (O.R.-S.); dtupayachy@ucsm.edu.pe (D.T.-Q.); 3 Escuela Profesional de Ingeniería Metalúrgica, Universidad Nacional de San Agustín de Arequipa, Calle Santa Catalina N°117 Cercado, Arequipa 04001, Peru; zhachire@unsa.edu.pe

**Keywords:** bioaccumulation, the *Buddleja* species, *Schinus molle* L., compost, total chromium

## Abstract

This research aimed to assess the bioaccumulation capacity of the *Buddleja* species and *Schinus molle* L. using organic amendments to the phytoremediation of total chromium in the mid-zone of the Añashuayco Ravine, Uchumayo, Arequipa, impacted by tanneries from the Rio Seco Industrial Park. Additionally, it analyzed total chromium concentrations, soil physicochemical properties, and morphological changes in plants with and without organic matter. Samples of the *Buddleja* species and *Schinus molle* L. were distributed into groups with and without compost, along with control groups. They were monitored over 6 months, every 60 days, showing significant morphological variations. The results highlight an important finding: the remarkable bioaccumulation capacity of the species studied all exceeded 30%. The samples without compost showed a lower percentage of total chromium bioaccumulation in plants compared to the samples with the organic amendment. The *Buddleja* species demonstrated a 39.01% chromium bioaccumulation with compost compared to 37.99% without it. Likewise, *Schinus molle* L. achieved 33.99% chromium accumulation with compost and 31.84% without it. These findings emphasize the superior ability of these species to bioaccumulate heavy metals, highlighting that the *Buddleja* species has mayor bioaccumulation capacity and more remotion of total chromium in the soil.

## 1. Introduction

In recent decades, tanneries have experienced a significant increase worldwide, especially in Europe and the United States since the 20th century. This is due to the growing demand for leather products in the fashion industry, driven by the need for industrialized materials [1]. The considerable demand has led to a notable increase in the generation of waste derived from this activity. This situation is attributed to the fact that the production of 1 t of processed leather requires 500 kg of chemical inputs, of which 85% are directly discarded into nearby water bodies and soil without prior treatment [1]. Additionally, various organic components, such as rawhide, fat, and hair, are removed during the leather production processes, generating both solid and liquid waste, combined with other organic and inorganic compounds [2].

Furthermore, the leather tanning process primarily uses Cr (III), which transitions to Cr (VI) through oxidation during tanning. Chromium and its forms are hardly biodegradable, making them an environmental burden due to their persistence, accumulation over time, and unpredictable effects. It is even more alarming that Cr (VI) is a proven carcinogen for humans [3].

In Peru, the leather industry has primarily developed in the cities of Trujillo, Arequipa, and Lima where new technologies are being implemented. [4]. These advancements include the use of automated machinery for tanning and processing hides and advanced quality control systems, as well as waste and wastewater treatment systems, thereby reducing the environmental impact of production [5]. However, only 50% of the leather produced nationally comes from formal companies, with the remaining 50% resulting from informal enterprises [6]. These informal enterprises produce untreated waste, posing a severe environmental and public health impact [6].

In the city of Arequipa, at the Rio Seco Industrial Park, where most tanneries are located, the area has become critical due to the high production of liquid and solid waste containing high levels of chemical residues, particularly hexavalent chromium. The problem lies in the fact that these effluents and wastes are discharged into two oxidation lagoons with a capacity of 28,000 m^3^, which quickly becomes overloaded due to the quantity of waste. Consequently, the wastewater flows into the environment without any treatment, impacting the surrounding areas [7]. It should also be noted that in many runoff points, the values of metals such as total chromium exceed the Peruvian Environmental Quality Standards (ECA in Spanish) for industrial-use soils [6], posing a significant risk to the environment, specifically soil resources and public health.

To reduce the amount of total chromium in the soil, various methods and technologies have been proposed in recent years, such as phytoremediation, which aims to reduce the concentration of potentially hazardous compounds through biochemical processes carried out by living systems [8]. Additionally, the effectiveness of different native plant species, such as Molle, Eucalyptus, Vilco, and Quinual, has been evaluated, demonstrating their capacity to bioaccumulate heavy metals [9].

It has also been shown that adding various elements to the bioaccumulation process, such as organic amendments, improves the treatment of soils contaminated with heavy metals, such as cadmium and lead [10]. These amendments can include animal manure, vermicompost, and biochar, among others [10]. Huaraca et al. [11] conclude that organic amendments (compost, vermicompost, etc.) function to increase binding sites, raise the soil’s hydrogen potential, form stable complexes with metals, and decrease the bioavailability of heavy metals, such as cadmium. Moreover, the use of organic amendments enhances soil properties, thereby boosting bioaccumulation and subsequently increasing soil remediation.

The present research aimed to evaluate the bioaccumulation capacity of the *Buddleja* species and *Schinus molle* L. using organic amendments for phytoremediation in the mid-section of the Añashuayco Ravine, located in the Uchumayo district of Arequipa. This area has been identified as impacted by residual effluent runoff from the overflow of oxidation ponds at the Rio Seco Industrial Park. The study focused on assessing the bioaccumulation potential of these species in soils contaminated with total chromium. Additionally, it involved determining the concentration of total chromium and the physicochemical parameters of soil samples from this section of the ravine at the beginning, during, and at the end of the experiment; evaluating morphological variations in the *Buddleja* species and *Schinus molle* L. through a qualitative visual assessment over the course of the study; analyzing total chromium bioaccumulation in both species by examining organic matter and applying a bioaccumulation percentage equation; and characterizing their bioaccumulative behavior in the presence of organic amendments, using organic matter content and bioaccumulation percentages as indicators.

## 2. Materials and Methods

### 2.1. Soil Sampling

The sampling area was located in the mid-section of the Añashuayco Ravine, where the residual effluent from the overflow of the Rio Seco Industrial Park’s oxidation lagoons flows freely. A single sampling point was chosen, taking into account the four sampling points from the study by Almirón et al. [12]. Of these, the highest concentration of total chromium in the soil was identified at a location near point P-Q1, which corresponds to the sampling point selected in this study.

**Figure 1:**
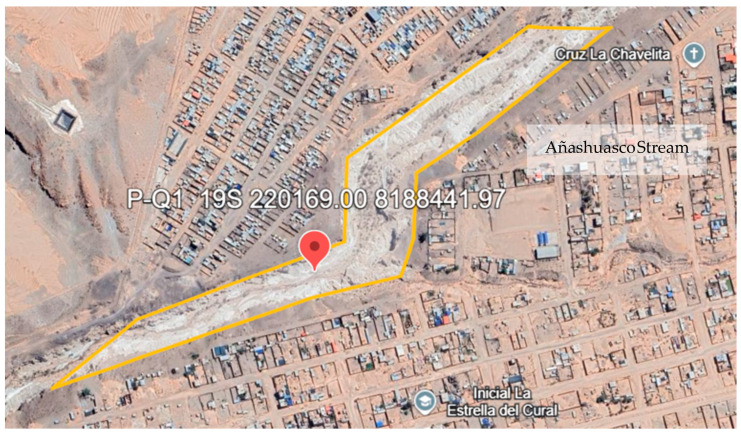
Location of the monitoring point in the evaluated area. Source: Own elaboration using Google Earth.

The location of point PQ-1 corresponds to the UTM coordinates WGS 84 (Zone 19K), East 220,169.00 m, and North 8,188,441.97 m. This location is shown in Figure 1.

For the sampling process, the guidelines provided by the ‘Soil Sampling Guide’ of the Ministry of the Environment of Peru were taken into account. This guide serves as a comprehensive framework for conducting soil sampling in potentially contaminated sites. It outlines essential methodologies, protocols, and standards necessary for effective soil assessment, ensuring that environmental regulations are executed [11]. Also, the guide encompasses various critical sections, including the objectives of soil sampling, planning and procedural guidelines, and detailed annexes [11]. It specifies the types of sampling methods, the necessary equipment, and the parameters to be analyzed, thereby providing a structured approach to soil investigation. In addition, the guide is pivotal in establishing standardized practices for soil sampling, which is crucial for environmental monitoring and remediation efforts. It assists in identifying contamination sources, evaluating the extent of pollution, and determining the effectiveness of remediation strategies [11].

Following the Soil Sampling Guide, it was first determined that the type of sampling would be identification-based [11]. 

The sampling pattern was random, as the study area is not uniform, and a sampling depth of 30 cm was used. At the sampling point, soil pits were excavated, the soil was mixed, and a single representative sample per sampling point was obtained. Soil samples were taken from the selected sampling point, and these samples were homogenized to obtain a representative sample per sampling point.

A sample of approximately 175 kg of soil was collected from the sampling point, and 400 g of the sample was sent to the laboratory for initial analysis. Then, the samples were divided into 48 pots, half of them with 4 kg of contaminated soil without compost and the other half containing approximately 2.7 kg of soil and 1.3 kg of compost. The amounts used were dry masses.

### 2.2. Cultivation of Buddleja Species and Schinus molle L.

The studied population consisted of two plant species, the *Buddleja* species and *Schinus molle* L, which were germinated in a nursery. The sample included 26 seedlings of the *Buddleja* species and 26 seedlings of *Schinus molle* L., which were used in the experimental tests and as controls.

In each pot, 4 kg of soil was placed for the transplantation of the *Buddleja* species and *Schinus molle* L. for tests without the addition of the stimulus (compost) and was categorized as BS and SS, respectively. In contrast, for tests with the addition of the stimulus (compost), the *Buddleja* species and *Schinus molle* L. were cataloged as BC and SC, respectively, and 2.7 kg of soil and 1.3 kg of compost were used in each pot (this amount is approximately 32% of the total), followed by the transplantation of the *Buddleja* species and *Schinus molle* L.

The samples comprised a total of 52 pots, divided into two groups for each species (24 pots each) in the following way: for the *Buddleja* species, they were distributed in subgroups of 4 pots each, of which 12 had compost, and 12 did not. For *Schinus molle* L., they were distributed in subgroups in the same way.

To select the soil and plant samples to be analyzed, it was determined that after 60 days, the subgroups ending in 1 would be extracted; after 120 days, the subgroups ending in 2 would be extracted; and after 180 days, the subgroups ending in 3 would be extracted. In this way, 3 containers were controlled every two months, and the subgroup ending in 4 served as a backup in case of some kind of mishap with any of the plant species. In addition, two control pots were placed for each group, one with compost and the other with uncontaminated soil. 

It was established that the pots should be watered every 48 h with 1.02 L, as determined from the calculations of the irrigation depth formula (based on the field capacity and wilting point). This volume was maintained during the 6 months of the experiment, and drinking water was used for watering according to the country’s quality standards. Additionally, a container was placed under each pot to collect the infiltrated water, which was used to water the seedlings and thus avoid the total loss of the chromium solution.

**Figure 2:**
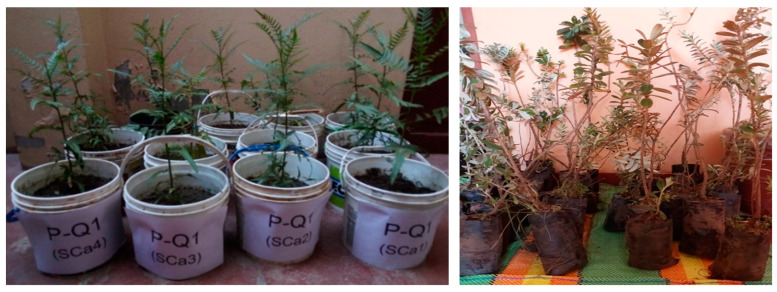
Plants tested.

The coding of the pots and the cultivation process can be found in Supplementary Materials. Figure 2 shows photographs of the experimental pots for both species. 

### 2.3. Evaluation of the Physicochemical Parameters of the Assessed Soil 

Considering that the soil–plant interface parameters are the primary control of metal absorption by the plant as they determine the processes of metal mobility and bioavailability [13], the most relevant physicochemical parameters evaluated in the soil samples were pH, conductivity, moisture, texture, organic matter, field capacity, wilting point, and irrigation depth. The conductivity and pH were measured at the beginning and end of the study to track variations as chromium was removed during phytoremediation, while other parameters were evaluated only at the beginning of the experiment. Moisture content was determined by oven-drying samples at 105 °C, while soil texture was classified by the hydrometer method to identify the sand, silt, and clay proportions. Organic matter was quantified through Loss on Ignition (LOI) analysis at 550 °C.

Field capacity and wilting point were measured via a Bar Pressure Plate Extractor, with field capacity set at −0.33 bars and wilting point at −15 bars to mark the threshold for plant water extraction. Irrigation lamina was calculated from evapotranspiration rates and soil properties to determine the necessary water application for optimal moisture.

Table 1 presents the laboratory results of the physicochemical parameter analysis of the initial soil.

### 2.4. Evaluation of the Total Chromium Concentration in Soil Samples

First, the initial total chromium analysis was performed using a representative soil sample extracted from the sampling point. Subsequently, representative samples were taken from each pot for analysis, except for the controls. Pots from Subgroup 1 were evaluated at 2 months; Subgroup 2 at 4 months; and finally, Subgroup 3 at 6 months.

The presence of total chromium in the soil was evaluated using EPA Method 3050B: Acid Digestion of Sediments, Sludges, and Soils, which is a protocol aimed at determining the total metal content, in this case by inductively coupled plasma mass spectrometry (ICP-MS). Using nitric and hydrochloric acids, it dissolves metals from organic matter and mineral matrices, offering a reliable estimate of environmentally available metals for regulatory assessments.

In accordance with the outlined methodology, the initial total chromium concentration in the soil was quantified at 2323 mg/kg. This value significantly exceeds the Peruvian Environmental Quality Standards (ECA) for soil, surpassing the threshold of 400 mg/kg for Category 2 (Residential/Park Soil) and 1000 mg/kg for Category 3 (Commercial/Industrial/Extractive Soil). 

### 2.5. Evaluation of the Morphological Characteristics of Buddleja Species and Schinus molle L.

The morphological characteristics of interest were determined, including plant height, leaf loss, and leaf color of the *Buddleja* species and *Schinus molle* L. These characteristics were evaluated every 60 days through data collection and photographs of the stems, leaves, and roots.

Daily monitoring was also conducted to observe variations in the physical structure or death of any seedlings, which may occur. Additionally, the possible presence of pests, viruses, or bacteria affecting the seedlings was controlled.

### 2.6. Determination of Total Chromium Bioaccumulation in Buddleja Species and Schinus molle L. Through Organic Matter Analysis and the Use of the Plant Bioaccumulation Percentage (%) Equation

The *Buddleja* species and *Schinus molle* L. initially contained no total chromium in their structure, as they were germinated in a nursery, resulting in an initial concentration of zero (0). To determine the bioaccumulation of each species, the following formula was used:

(1) $$\%\;\mathrm{of}\;\mathrm{Plant}\;\mathrm{Bioaccumnulation}=\frac{\mathrm{Cc}\;\mathrm{of}\;\mathrm{Total}\;\mathrm{Cr}\;\mathrm{in}\;\mathrm{the}\;\mathrm{Plant}\;\mathrm{at}\;\mathrm{the}\;\mathrm{End}\;\mathrm{of}\;\mathrm{the}\;\mathrm{Experiment}\;\times 100}{\mathrm{Cc}\;\mathrm{of}\;\mathrm{Total}\;\mathrm{Cr}\;\mathrm{in}\;\mathrm{the}\;\mathrm{Soil}\;\mathrm{at}\;\mathrm{the}\;\mathrm{Beginning}\;\mathrm{of}\;\mathrm{the}\;\mathrm{Experiment}}$$

Since the initial concentration in the plant was zero, and the final concentration was determined through laboratory analysis, each seedling was removed from each sampling point every 60 days and at the end of the experiment. The plant was left to dry in the sun (stems, leaves, and roots) to determine the percentage of total chromium bioaccumulated for the analysis. However, only the samples collected at the end of the experiment were sent to the laboratory for analysis and determination of total chromium concentration in the seedlings. This was performed using atomic absorption spectrophotometry, hydride generation, and cold vapor techniques. 

At the end of the research, the amount of total chromium absorbed by the plant was determined. Additionally, by obtaining the final contaminant levels in both the soil and the plant, it was assessed whether there was any loss of chromium during the process and if the metal was properly absorbed by the plant.

### 2.7. Statistical Analysis

All experiments were performed in triplicate to ensure both the accuracy and reproducibility of the results. The values presented in certain tables and figures represent the mean and standard error calculated from the triplicates.

The statistical analysis of the results found was carried out using the IBM SPSS Statistics test package, using analysis of variance.

## 3. Results and Discussions

### 3.1. Physicochemical Properties of the Initial Soil Sample

According to the initial pH analysis (7.30), it was close to neutrality. Cr (III) is more strongly adsorbed by sandy soils; however, this reaction is pH-dependent. A higher pH increases the adsorption of Cr (III), while Cr (VI) adsorption decreases at pH values around 8.5 [13], making it more soluble, mobile, and bioavailable, allowing for its uptake by plant roots. A higher pH increases the adsorption of Cr (III), while Cr (VI) adsorption decreases at pH values around 8.5 [13], making it more soluble, mobile, and bioavailable, allowing for its uptake by plant roots [14].

Its conductivity was 3.29 mmho/cm, indicating a saline character that could potentially affect the growth of the seedlings. The soil was loamy sand, facilitating the movement of the irrigation water and the growth of the roots. The organic matter content was extremely low, which could impact the growth of the seedlings.

The field capacity was 9.20%, the wilting point was 1.88%, and the irrigation requirement for the crops was 1.02 L every 48 h.

**Figure 3:**
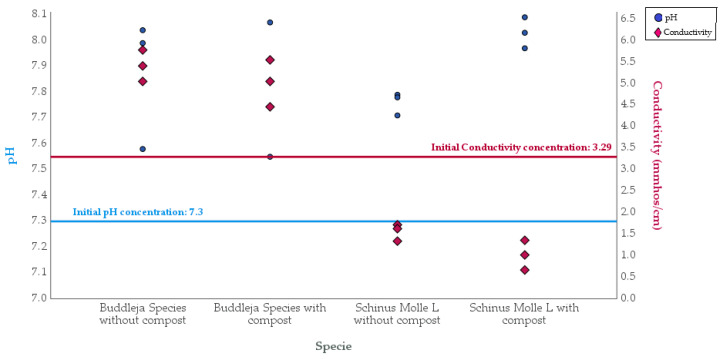
pH and conductivity of the sampled soil during experimentation (0 days and 180 days).

Additionally, the physicochemical parameters of pH and conductivity were evaluated at the end of the study to verify the variations that occurred due to phytoremediation for both *Schinus molle* L. and the *Buddleja* species, as shown in Figure 3, and additional data are in Supplementary Materials.

The initial pH of the sample was 7.3, which falls within the neutral range. At the end of the experimentation, both the *Buddleja* species and *Schinus molle* L. showed an increase in pH, although the variation was insignificant. The *Buddleja* species fell into the moderately alkaline soil category, while *Schinus molle* L. was in the moderately basic range. In this pH range, Cr (III) adsorption is above 60% [15].

The initial electrical conductivity was 3.29 mmho/cm and was categorized as very slightly saline, which is normal for plant growth [16]. For the *Buddleja* species, the electrical conductivity ranged from 4.46 mmho/cm to 5.78 mmho/cm and was classified as slightly saline, which is suitable for the growth of the *Buddleja* species. Conversely, *Schinus molle* L. was in the range of 0.67 mmho/cm to 1.79 mmho/cm and considered non-saline and appropriate for plant growth.

At the end of the experimental process, the pH and conductivity of the soil samples were analyzed, obtaining the following results from the analysis of variance (ANOVA) and the Eta-squared, which are shown in Table 2 and Table 3.

In the case of pH, this obtained a significance of 0.539, which is greater than 0.01, indicating that there are no statistically significant differences in the pH of the samples at the end of the experiment. That is to say, the treatment applied did not have a relevant effect on the pH of the soil, and regarding the eta-squared, this had a value of 45.1%, indicating that the variation in the pH values could be explained by the treatment applied. This percentage suggests that the pH presents a certain sensitivity to the conditions of the experiment, although the differences are not sufficiently marked to be considered significant.

Regarding conductivity, it obtained a significance less than 0.01, a value that indicates that there is a statistically significant difference in the conductivity between the samples analyzed at the end of the process. Therefore, the applied treatment had a clear impact on soil conductivity, and as for eta squared, it had a value of 98.3%, which is an extremely high value that can be attributed to the applied treatment, demonstrating that conductivity is a variable highly influenced by the experimental conditions. In addition, the difference in conductivity could have influenced the mobility of nutrients and chromium, making it more bioavailable for the *Buddleja* species and less bioavailable for *Schinus molle* L.

### 3.2. Morphological Variations in the Evaluated Species

**Figure 4:**
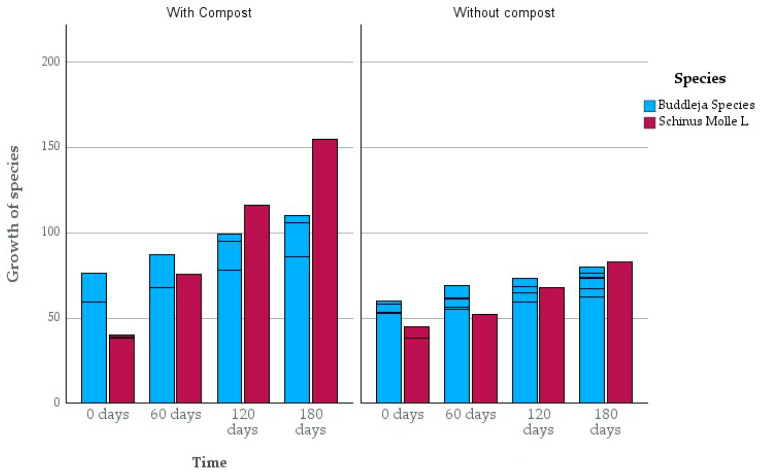
Growth of species during experimentation. The black lines indicate the value of size of each subgroup, whose averages are found in Table 4.

During experimentation, data on plant height, leaf color, and leaf loss were recorded from day 0, followed by every two months. Likewise, the growth values of both species with and without the presence of compost were recorded, taking into account the controls, and are shown in Table 4 and Figure 4. 

The growth of the species is reflected in the difference in size (cm) at the beginning (0 days) and end of the experiment (180 days). It can be observed that the growth of the control without compost BBTh was around 35 cm, while the samples with compost grew, on average, 31.19 cm, and the samples without compost grew, on average, 15.9 cm. Regarding *Schinus molle* L., the results were the following: the growth of the control without compost SBTh was 168 cm, while the samples with compost grew, on average, 58.25 cm, and the samples without compost grew, on average, 30.12 cm. 

This behavior for both species is clearly reflected in Figure 4, where the difference in growth between plants with and without compost can be seen. This is because the organic matter in the compost improves soil structure and water retention, maintaining better conditions for the study species. In addition, it was verified that the color of the species was dark green, and they did not present signs of disease on the leaves and/or stems.

Finally, according to Table 5, it can be seen that the significance value of the ‘Species’ group is greater than 0.05, so this factor does not have a significant effect on the dependent variable ‘Size’. As for the interactions ‘Species*Treatment’, ‘Species*Time’, and ‘Species*Treatment*Time’, their *p*-values less than 0.05 indicate that the effect of one factor depends on the level of the other, which is significant and demonstrates an important impact on the ‘Size’ variable.

### 3.3. Total Chromium Concentration in Soils During and After Experimentation with Plant Species 

Table 6 shows the concentration of the contaminated soil analyzed at 60, 120, and 180 days during the experiment. At the end of the experimental tests, samples were subdivided into groups and subgroups according to the presence of compost in the soil. 

In group ‘a’, it was observed that both the *Buddleja* species and *Schinus molle* L. showed a decreasing trend in total chromium concentration (mg/kg) in the evaluated samples. It was determined that the values found at 60 days for the *Buddleja* species were 2217 mg/kg for BSa1 and 2187 for BCa1, which reduced to 1408 mg/kg and 1093 mg/kg, respectively, at 180 days. For *Schinus molle* L., the same behavior was observed, with values identified at 60 days being 1909 mg/kg for SSa1 and 2268 for BCa1, which reduced to 1441 mg/kg and 1155 mg/kg, respectively, at 180 days. Thus, with and without compost, both species exhibited a high capacity for the phytoremediation of soil contaminated with total chromium, reducing more than half of the initial concentration. Almirón et al. [12] demonstrated that the *Buddleja* species can reduce total chromium (mg/kg) in contaminated soil from 1784.44 mg/kg to 1042.89 mg/kg over 90 days. Additionally, it should be noted that chromium concentrations were lower in the presence of compost compared to samples without it.

In group ‘b’, it was observed that both the *Buddleja* species and *Schinus molle* L. exhibited a decreasing trend in total chromium concentration (mg/kg) in the evaluated samples. It was determined that the values found at 60 days for the *Buddleja* species were 2315 mg/kg for BSb1 and 1923 for BCb1, which reduced to 1389 mg/kg and 1113 mg/kg, respectively, at 180 days. For *Schinus molle* L., the same behavior was observed, with values identified at 60 days being 1951 mg/kg for SSb1 and 2271 for BCb1, which reduced to 1239 mg/kg and 1526 mg/kg, respectively, at 180 days. Both species exhibited a high capacity for the phytoremediation of soil contaminated with total chromium, reducing by more than 500 mg/kg in the first species and more than 800 mg/kg in the second species. Paredes et al. [9] demonstrated that *Buddleja Coriacea* can achieve reduction percentages of over 80% in heavy metals such as antimony, arsenic, cadmium, copper, silver, and lead. Similarly, *Schinus molle* L. exhibited reduction percentages of up to 67.8% for heavy metals such as arsenic, copper, and lead. Unlike group ‘a’, in group ‘b’, only the *Buddleja* species maintained the relationship of a lower chromium concentration in the presence of compost, as *Schinus molle* L. showed a lower concentration without compost.

In group ‘c’, it was observed that both the *Buddleja* species and *Schinus molle* L. showed a decreasing trend in the total chromium concentration (mg/kg) in the evaluated samples. It was determined that the values found at 60 days for the *Buddleja* species were 1530 mg/kg for BSc1 and 2055 for BCc1, which reduced to 785 mg/kg and 968 mg/kg, respectively, at 180 days. For *Schinus molle* L., the same behavior was observed, with values identified at 60 days being 1770 mg/kg for SSc1 and 1730 for BCc1, which reduced to 1244 mg/kg and 1338 mg/kg, respectively, at 180 days. Both species exhibited a high capacity for the phytoremediation of soil contaminated with total chromium, reducing by more than 600 mg/kg in the first species and more than 1000 mg/kg in the second species. Waranusantigul et al. [16] demonstrated that *Buddleja asiatica* and *Buddleja paniculata* could accumulate up to 3675 mg/kg of lead (Pb) in their shoots over 6 months, i.e., 180 days. Unlike the previous groups, in group ‘c’, the chromium concentrations were lower in the absence of compost compared to samples with it. 

According to Table 7, the significance value of the ‘Concentration*Species’ interaction is greater than 0.05, indicating that this interaction does not have a significant effect on the dependent variable ‘Concentration’. On the other hand, the Eta-squared analysis reveals that differences between species explain 6% of the total variability in the soil chromium concentration. This value of 0.06 suggests that phytoremediation species have a statistically moderate impact on soil chromium bioaccumulation processes.

It should be noted that although plant species have the inherent ability to bioaccumulate or extract chromium from the soil, this effectiveness can be affected by soil conditions and the addition of organic amendment. During experimentation, soil conductivity was increased, so the bioavailability of chromium could have been influenced by this. Also, the pH was between 7 and 8, and according to Han (2004), an acidic pH is more favorable for the solubilization of the heavy metal and its bioavailability for plants [14].

Likewise, the added organic amendment could have influenced the interaction of chromium with plant species, modifying the physicochemical properties of the soil. This is because organic amendments often contain compounds that can chelate heavy metals, forming stable complexes that reduce the bioavailability of chromium. This effect, combined with a less favorable pH, could have limited the plants’ ability to take up chromium [13]. On the other hand, it is also possible that the amendment has positively influenced the development of the plants by improving the quality of the soil [11]; even so, it has not been sufficient to counteract the limitations imposed by the pH of the experiment.

### 3.4. Bioaccumulation Capacity of Total Chromium in the Evaluated Plant Species

The laboratory results are shown in Table 8, with laboratory tests conducted at the end of the experimentation (180 days). The bi-monthly removal of experimental pots was performed, and along with the extraction of soil from each subgroup, the seedlings of each species were carefully removed separately for the *Buddleja* species and *Schinus molle* L. Additionally, the percentage of bioaccumulation of the plant was calculated relative to the initial soil concentration using the Formula (1) in Section 2.6, which was used for both species.

Regarding the standard error of the concentration in the case of the *Buddleja* species without compost, it is ±3.5; for the *Buddleja* species with compost, it is ±4.6. In the case of *Schinus molle* L., the deviation for the samples without compost is ±7.3, and for the samples with compost, it is ±3.8. The presence of compost has opposite effects on the two species: it increases the variability in concentration measurements for the *Buddleja* species but reduces it for *Schinus molle* L. 

**Figure 5:**
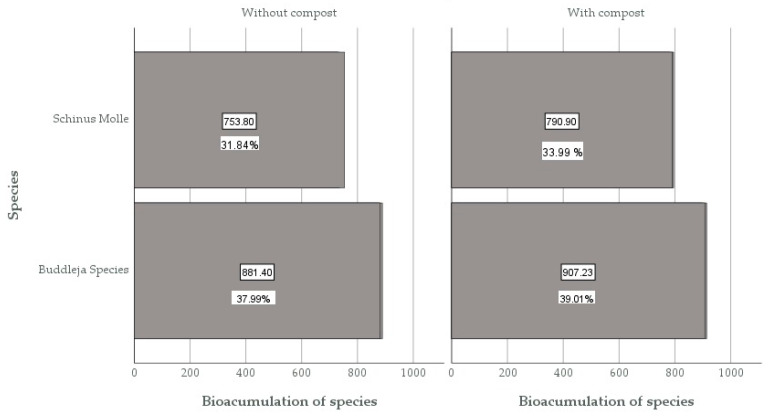
Comparison of % bioaccumulation of the species at the end of the evaluation.

In Figure 5, it can be observed that the bioaccumulation percentage (%) of both species exceeded 30%, indicating that they are a viable option for use in phytoremediation techniques. The highest value was for the *Buddleja* species, with 39.01% bioaccumulation compared to 33.04% for *Schinus molle* L. The highest bioaccumulation value was in the BCb3 sample of the *Buddleja* species compared to its counterpart *Schinus molle* L., with 795.6 mg/kg. This is evidenced by the investigation of Salas et al. [17], where *Buddleja scordioides* bioaccumulated lead (Pb) at 1378 ug/g in its stem, making it a candidate for the phytoremediation of soils contaminated with the heavy metal lead.

The bioaccumulation percentage (%) of both species exceeded 30%, indicating they are viable for use in phytoremediation techniques, with the *Buddleja* species having the highest value at 37.99% compared to 31.84% for *Schinus molle* L. The highest bioaccumulation value was observed in the BSb3 sample of *Buddleja* species compared to its counterpart *Schinus molle* L., with 753.8 mg/kg. This is evidenced by the investigation of Rodríguez [18], where *Buddleja cordata* can be used to bioaccumulate hydrocarbons (BTEX) and Total Petroleum Hydrocarbon fractions, demonstrating the species’ tolerance to environmental stress. Likewise, according to Zhang et al. [19], the *Buddleja* species has a greater bioaccumulation of Cr compared to other native species, and this bioaccumulation occurs noticeably more in their leaves and stems than in their roots, which indicates the capability of *Buddleja* Species to translocate Cr from their roots to their shoots.

It was observed that samples without compost showed a lower bioaccumulation percentage (%) of total chromium compared to samples with organic amendments [20]. It was also determined that there is 39.010% metal removal from the soil for the *Buddleja* species with compost and 37.99% for samples without compost. Similarly, for *Schinus molle* L., there is 33.994% removal with compost and 31.836% without compost, demonstrating that compost aided in heavy metal removal. This is consistent with Liu et al. [20] and Rodrigo [21], demonstrating that the addition of organic amendments enhances the phytoremediation capacity of species for soils contaminated with chromium and lead. This bioaccumulation is due to organic amendments contributing to the solubilization of heavy metals such as chromium, lead, and cadmium in the soil, according to Munive et al. [22]. The percentage difference is also due to species differences, as *Schinus molle* L. seedlings have shown greater bioaccumulation of heavy metals, as shown by Paredes et al. [9], achieving higher removal percentages for arsenic, lead, and copper, and surpassing these percentages by more than 30% for lead (Pb).

The statistical analysis of the bioaccumulation percentages of the species at the end of the experimentation, presented in Table 9, reveals different patterns in the responses of the studied species. Among them, *Schinus molle* L. without compost stood out as the species with the greatest dispersion in the data, suggesting highly heterogeneous responses to the experimental conditions. This variability could be due to individual differences between plants, their adaptability to changes in the soil, or limitations in the bioaccumulation capacity under specific conditions, such as the absence of organic amendment [11].

A relevant aspect is that although *Schinus molle* L. presented a higher growth rate compared to the *Buddleja* species, it showed a lower bioaccumulation of chromium. This behavior could be related to the physiological limitations that plants face when accumulating high concentrations of heavy metals. It is known that the storage of high levels of chromium in tissues can negatively affect fundamental processes such as regenerative capacity, enzymatic activity, integrity of the membrane structure, and electron mobility, compromising cellular functionality [23].

In addition, signs of physiological stress were observed in both species, such as the discoloration of the leaves and their slight fall. These symptoms are common indicators of heavy metal toxicity, resulting from damage to the photosynthetic and metabolic systems, as well as alterations in cellular homeostasis. On the contrary, although the *Buddleja* species had more limited growth, its greater bioaccumulation of chromium could reflect a greater tolerance to the toxicity of the metal or a more efficient mechanism for its storage despite the cost in terms of biomass [12].

Finally, the significance analysis of the ‘Concentration*Species’ interaction, with a value less than 0.01, reinforces the idea that the differences between species are not random but rather respond to specific interactions between chromium concentrations in the soil and the physiological characteristics of each plant. This highlights that phytoremediation strategies must consider not only the bioaccumulation capacity but also the impact of the heavy metal on the vitality and health of the species used [23].

## 4. Conclusions

According to the variance analysis, it can be inferred that the removal of total chromium is not influenced by pH, but it is influenced by the conductivity of the soil. This explains why the Buddleja species, having a higher conductivity, also presents a higher chromium bioaccumulation capacity. 

Also, it is evident that the removal of chromium in soils is directly proportional to the passage of time, i.e., the higher the percentage of removal efficiency (%) in the soil. Also, *Schinus molle* L. presented a higher growth rate compared to the *Buddleja* species and showed a lower bioaccumulation of chromium. This indicates that the differences between the two species are due to specific interactions between chromium concentrations in the soil and the physiological characteristics of each plant.

It was demonstrated that the use of organic amendments does not have an effect on the bioaccumulation of total chromium in the evaluated species, especially in the *Buddleja* species, which presented a higher percentage of bioaccumulation in comparison to *Schinus molle* L.

In conclusion, the *Buddleja* species and *Schinus molle* L. were able to reduce total chromium in contaminated soils due to their ability to bioaccumulate total chromium in their roots, stems, and foliage. This ability has the remarkable results of surpassing 30% bioaccumulation by both species.

In further research, it is advisable to use different percentages of amendments to evaluate the optimal percentage for both plant growth and the phytoremediation capacity of the species.

## Figures and Tables

**Table 1:** Physicochemical parameters of soil.

Parameter	Result	Unit
Concentration of Total Chromium	2323	mg/kg
Ph	7.30	pH
Conductivity	3.29	mmho/cm
Texture	Clay 4.8	%
	Sand 79.2	%
	Silt 16.0	%
Organic Matter	0.17	%
Textural Class	Loamy Sand	---
Moisture	16.97	%
Field Capacity	9.20	%
Wilting Point	1.88	%
Irrigation Lamina	1.02	L

**Table 2:** Analysis of variance (ANOVA) for pH and conductivity.

		ANOVA				
		Sum of Squares	df	Mean Square	F	Sig.
pH	Between Groups	0.130	3	0.043	0.777	0.539
	Within Groups	0.447	8	0.056		
	Total	0.577	11			
Conductivity	Between Groups	46.936	3	15.645	106.179	<0.001
	Within Groups	1.179	8	0.147		
	Total	48.115	11			

**Table 3:** ANOVA effect size ^a,b^.

	Group	Point Estimation	Mean Square	F
pH	Eta-squared	0.226	0.000	0.451
	Epsilon-squared	−0.065	−0.375	0.245
	Omega–squared fixed effect	−0.059	−0.333	0.229
	Omega–squared random effect	−0.019	−0.091	0.090
Conductivity	Eta-squared	0.976	0.870	0.983
	Epsilon-squared	0.966	0.821	0.977
	Omega–squared fixed effect	0.963	0.808	0.975
	Omega–squared random effect	0.898	0.584	0.929

Note: ^a^ Eta-squared are estimated based on the fixed effect. ^b^ Negative but less biased estimates are retained and not rounded to zero.

**Table 4:** Growth of the species during experimentation.

Subgroup	0 Days (cm)	60 Days (cm)	120 Days (cm)	180 Days (cm)
BCa	62.80 ± 4.22	73.75 ± 3.66	84.80 ± 5.27	95.53 ± 5.17
BCb	55.53 ± 7.50	66.75 ± 6.91	76.60 ± 6.57	87.33 ± 6.64
BCc	48.58 ± 3.88	57.35 ± 3.90	66.50 ± 3.88	77.63 ± 2.99
BSa	52.68 ± 2.70	63.05 ± 2.03	68.08 ± 2.30	74.30 ± 2.35
BSb	54.70 ± 1.02	59.33 ± 1.03	64.93 ± 1.18	72.33 ± 0.89
BSc	54.68 ± 1.18	57.40 ± 1.32	60.53 ± 1.53	63.13 ± 1.39
BBCg *	55.9	58	61.1	63.5
BBTh *	50	61.2	78.1	85
SCa	37.25 ± 1.80	67.00 ± 2.35	98.75 ± 5.41	129.50 ± 8.63
SCb	31.5 ± 2.66	53.50 ± 2.75	77.50 ± 3.33	100.50 ± 4.19
SCc	36.75 ± 0.63	68.00 ± 3.81	101.25 ± 7.42	133.50 ± 11.05
SSa	30.00 ± 4.64	40.25 ± 4.03	52.50 ± 3.57	63.75 ± 3.33
SSb	36.00 ± 2.35	44.50 ± 2.47	55.00 ± 2.92	64.50 ± 3.57
SSc	38.63 ± 2.21	47.00 ± 2.68	57.38 ± 4.09	66.75 ± 5.79
SBCg *	26	59	94	128
SBTh *	35	90	147	203

Note: Values show the mean of four replicates ± standard errors. * Blanks did not present more than one repetition, so there are no mean or standard errors.

**Table 5:** Between-subject effects (ANOVA) of species growth and days of experimentation.

Test of Between-Subjects Effects					
Dependent Variable: Size					
Source	Type III of Sum of Squares	df	Mean Square	F	Sig.
Corrected Model	85,367.209 ^a^	15	5691.153	55.485	<0.001
Intersection	816,134.481	1	816,134.481	7956.836	<0.001
Species	373.804	1	373.804	3.644	0.058
Between Groups	16,633.992	1	16,633.992	162.172	<0.001
Treatment	44,551.164	3	14,850.388	144.783	<0.001
Time	4436.169	1	4436.169	43.250	<0.001
Species*Treatment	8173.746	3	2724.582	26.563	<0.001
Species*Time	8466.061	3	2822.020	27.513	<0.001
Species*Treatment*Time	2732.354	3	910.785	8.880	<0.001
Error	18,052.359	176	102.570		
Total	919,554.130	192			
Corrected Total	103,419.649	191			

Note: ^a^ R-squared = 0.825 (adjusted R-squared = 0.811).

**Table 6:** Total chromium concentration in soil samples with and without compost.

Species	Without Compost		With Compost	
	Subgroup	Concentration (mg/kg)	Subgroup	Concentration (mg/kg)
*Buddleja*	BSa1	2217	BCa1	2187
	BSa2	1600	BCa2	1700
	BSa3	1408	BCa3	1093
	BSb1	2315	BCb1	1923
	BSb2	1676	BCb2	1600
	BSb3	1389	BCb3	1113
	BSc1	1530	BCc1	2055
	BSc2	1000	BCc2	1038
	BSc3	785	BCc3	968
*Schinus molle* L.	SSa1	1909	SCa1	2268
	SSa2	1823	SCa2	1853
	SSa3	1441	SCa3	1155
	SSb1	1951	SCb1	2271
	SSb2	1599	SCb2	1980
	SSb3	1239	SCb3	1526
	SSc1	1770	SCc1	1730
	SSc2	1434	SCc2	1866
	SSc3	1244	SCc3	1338

**Table 7:** Analysis of variance (ANOVA) for interactions between total chromium concentration and species with and without compost.

Report						
Concentration						
Species	Average	N	Standard Deviation	Standard Mean Error	Asymmetry	
*Buddleja* species Without Compost	1546.6667	9	497.82025	165.94008	0.189	
*Buddleja* species With Compost	1519.6667	9	476.61934	158.87311	0.146	
*Schinus molle* L. Without Compost	1601.1111	9	275.49839	91.83280	−0.108	
*Schinus molle* L. With Compost	1776.3333	9	384.84315	128.28105	−0.255	
Total	1610.9444	36	412.35137	68.72523	−0.060	
	**ANOVA**					
		**Sum of Squares**	**df**	**Mean Square**	**F**	**Sig.**
Concentration*Species	Between Groups	359,221.000	3	119,740.333	0.685	0.568
	Within Groups	5,591,956.889	32	174,748.653		
	Total	5,951,177.889	35			
**Measure Association**						
	**Eta**	**Eta-squared**				
Concentration*Species	0.246	0.060				

**Table 8:** Total chromium concentration in plants with and without compost.

Species	Group	Concentration (mg/kg)	* Concentration Average (mg/kg)
*Buddleja* species	BSa3	877.44	882.71 ± 3.5
	BSb3	889.3	
	BSc3	881.4	
	BCa3	897.7	906.21 ± 4.6
	BCb3	913.7	
	BCc3	907.23	
*Schinus molle* L.	SSa3	735.44	739.56 ± 7.3
	SSb3	729.44	
	SSc3	753.8	
	SCa3	795.6	789.68 ± 3.8
	SCb3	782.55	
	SCc3	790.9	

Note: * Values show the mean of three replicates ± standard errors.

**Table 9:** Analysis of variance (ANOVA) of % bioaccumulation of the species at the end of the evaluation.

Report						
Concentration						
Species Average		Average	N		Standard Deviation	
*Buddleja* species Without Compost		882.7133	3		6.03809	
*Buddleja* species With Compost		906.2100	3		8.04862	
*Schinus molle* L. Without Compost		739.5600	3		12.69186	
*Schinus molle* L. With Compost		789.6833	3		6.60953	
Total		829.5417	12		71.21284	
	ANOVA					
		**Sum of Squares**	**df**	**Mean Square**	**F**	**Sig.**
Concentration*Species	Between Groups	55,171.940	3	18,390.647	240.394	<0.001
	Within Groups	612.016	8	76.502		
	Total	55,783.955	11			
**Measure Association**						
	**Eta**	**Eta-squared**				
Concentration*Species	0.994	0.989				

## Data Availability

The original contributions presented in the study are included in the article/Supplementary Materials, further inquiries can be directed to the corresponding author.

## Supplementary Materials

The following supporting information can be downloaded at: https://www.mdpi.com/article/10.3390/plants13243469/s1.
