# Supplementary material for: Bioaccumulation of Cr by the Buddleja Species and Schinus molle L. Grown with and Without Compost in a Sandy Soil Contaminated by Leather Industrial Effluents
Source: Plants (Basel). 2024 Dec 11;13(24):3469. doi: 10.3390/plants13243469 (PMC11728836; doi:10.3390/plants13243469)
Supplement: Supplementary file 1 [file plants-13-03469-s001.zip › plants-3262954-supplementary.pdf]

Supporting Information for:

Article

Bioaccumulation of Cr by the *Buddleja* Species and *Schinus molle* L. Grown with and Without Compost in a Sandy Soil Contaminated by Leather Industrial Effluents

Jamilet Huarsaya-Huillca [1], Sheyla Callo-Sánchez [1], Camila Aguilar-Ccuno [1], Oswaldo Rodríguez-Salazar [2], Danny Tupayachy-Quispe [2], Giuliana Romero-Mariscal [1], Zulema Hachire-Patiño [3] and Jonathan Almirón [2,*]

[1] Escuela Profesional de Ingeniería Ambiental, Universidad Nacional de San Agustín de Arequipa, Calle Santa Catalina N°117 Cercado, Arequipa 04001, Peru; jhuarsayah@unsa.edu.pe (J.H.-H.); scallos@unsa.edu.pe (S.C.-S.); caguilarcc@unsa.edu.pe (C.A.-C.); gromeroma@unsa.edu.pe (G.R.-M.)
[2] Laboratorio de Ciencia de los Materiales, Facultad de Ciencias e Ingenierías Físicas y Formales, Universidad Católica de Santa María, Samuel Velarde 320, Arequipa 04000, Peru; orodriguez@ucsm.edu.pe (O.R.-S.); dtupayachy@ucsm.edu.pe (D.T.-Q.)
[3] Escuela Profesional de Ingeniería Metalúrgica, Universidad Nacional de San Agustín de Arequipa, Calle Santa Catalina N°117 Cercado, Arequipa 04001, Peru; zhachire@unsa.edu.pe
* Correspondence: jalmiron@ucsm.edu.pe; Tel.: +54-950000426

Number of Pages: 8

SI Figures: 04

SI Tables: 03

Table of Contents

1. Coding of experimentation pots
2. Photographs before and during the experimentation

SI Section 1. Coding of experimentation pots

Pots were coded according to the presence of compost, contaminated soil and plant species. The order of coding is exemplified in Table S1, the first column indicates the species (B and S), the second column indicates the presence of compost (S and C) and the control samples (BB for *Buddleja Species and* BS for *Schinus Molle* L.), also Th (with compost) and Cg (without compost), the third column indicates the group (a, b and c) and the fourth column indicates the subgroup (1, 2, 3 and 4). These are detailed in Table S1.

Table S1. Order and meaning of codes

Column	Type	Meaning	Code
**First Column**	**Species**	*Buddleja Species*	B
		Schinus Molle L.	S
**Second Column**	**Presence of compost**	With compost	C
		Without compost	S
	**Blank or not blank**	Field blank (exception)	BBTh, BBCg and SBTh SBCg
**Third Column**	**Group**	Group 1	a
		Group 2	b
		Group 3	c
**Fourth Column**	**Subgroup**	Subgroup 1	1
		Subgroup 2	2
		Subgroup 3	3
		Subgroup 4	4

This coding was used through the experimentation, which is detailed in Table S2.

Table S2. Pot coding for study.

**Species**	**Without compost**		**With compost**	
	**Group**	**Subgroup**	**Group**	**Subgroup**
	BSa	BSa1	BCa	BCa1

		BSa2		BCa2
		BSa3		BCa3
		BSa4		BCa4
Buddleja Species	BSb	BSb1	BCb	BCb1
		BSb2		BCb2
		BSb3		BCb3
		BSb4		BCb4
	BSc	BSc1	BCc	BCc1
		BSc2		BCc2
		BSc3		BCc3
		BSc4		BCc4
	BBTh	Control	BBCg	Control
Schinus Molle L	SSa	SSa1	SCa	SCa1
		SSa2		SCa2
		SSa3		SCa3
		SSa4		SCa4
	SSb	SSb1	SCb	SCb1
		SSb2		SCb2
		SSb3		SCb3
		SSb4		SCb4
	SSc	SSc1	SCc	SCc1
		SSc2		SCc2
		SSc3		SCc3
		SSc4		SCc4
	SBTh	Control	SBCg	Control

Table S3: *pH and conductivity of the soil sampled during the experiment.*

Species		Group	Subgroup	pH	Conductivity (mmhos/cm)
Buddleja Species	With compost	BCa	BCa3	8.32	4.46
		BCb	BCb3	8.07	5.05
		BCc	BCc3	7.55	5.55
	Without compost	BSa	BSa3	7.58	5.78
		BSb	BSb3	7.99	5.05
		BCc	BSc3	8.04	5.41
Schinus Molle L.	With compost	SCa	BCa3	7.97	1.36
		SCb	BCb3	8.09	0.67
		SCc	BCc3	8.03	1.02
	Without compost	SSa	BSa3	7.79	1.72
		SSb	BSb3	7.71	1.63
		SSc	BSc3	7.78	1.34

SI Section 2. Photographs before and during the experimentation

The research included field work including exploration of the area, initial monitoring of the sampled soil, planting of the species and monitoring during and at the end of the experiment. Photos have been taken during these stages which are shown in Figure S1, S2, S3, S4 and S5.

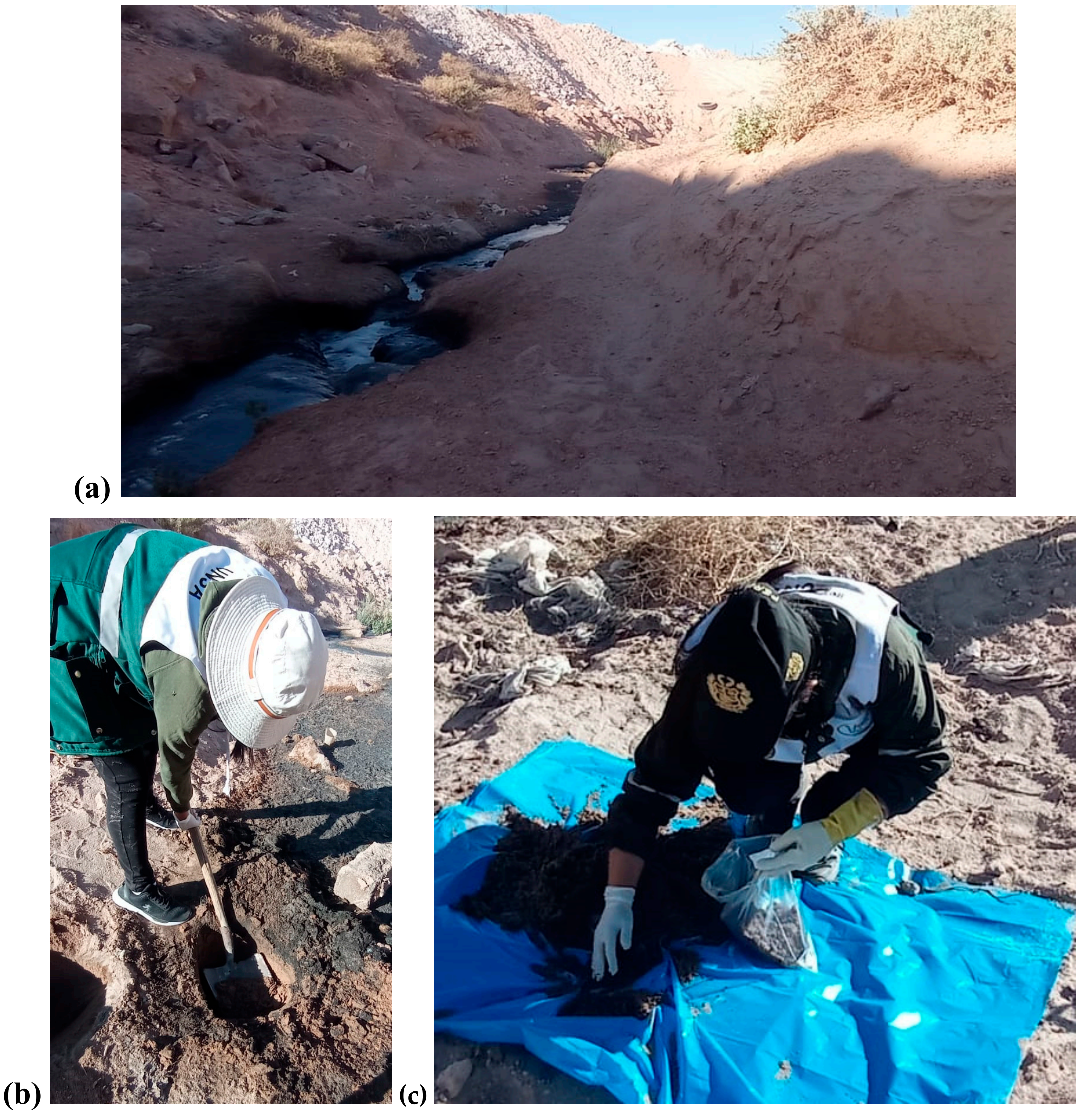

(a) (b) (c)

Figure S1. Field photos of (a) tannery effluent; (b) extraction of contaminated soil for initial analysis; (c) application of the soil sampling methodology established by the Peruvian Ministry of the Environment (Ministerio del Ambiente (MINAM) del Perú) to take the sample to the laboratory for analysis.

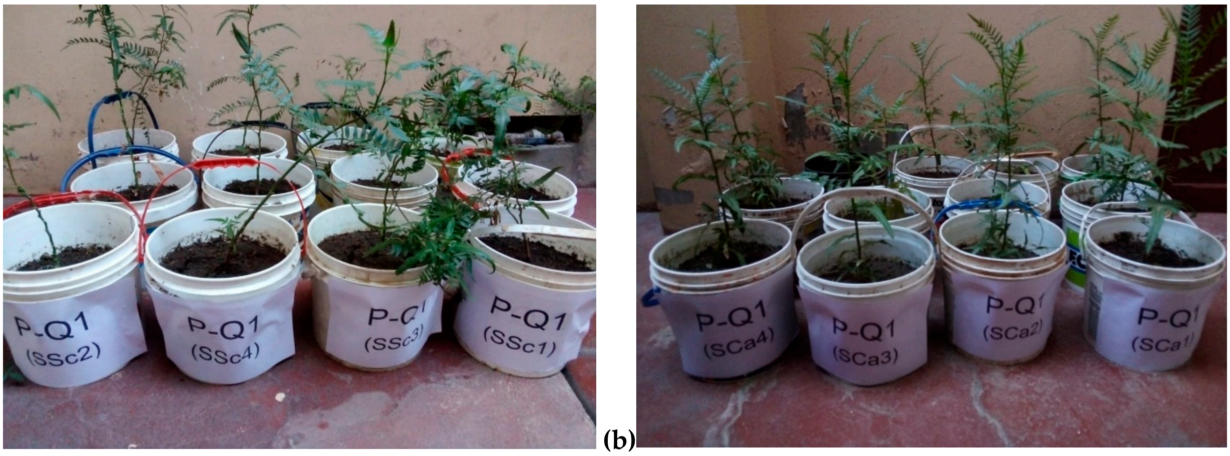

(a) (b)

(c) 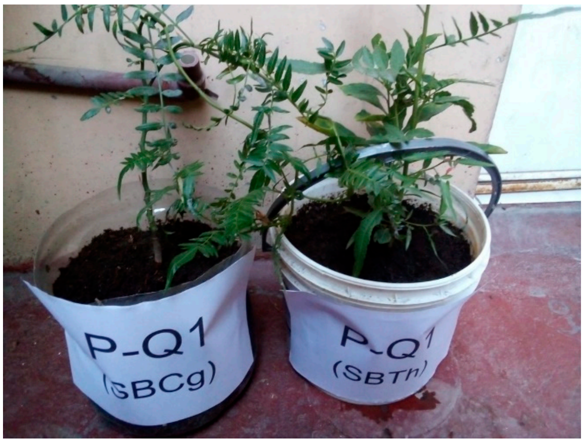

Figure S2. Photos of *Schinus Molle* L. in (a) pots without compost; (b) pots with compost and (c) pots of control samples.

(a) 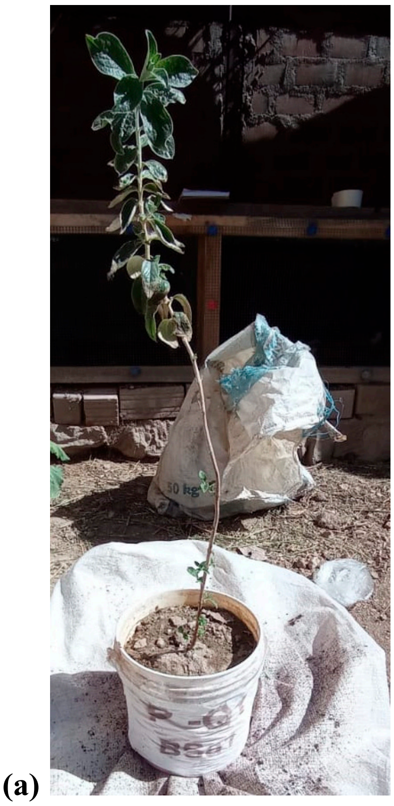

(b) 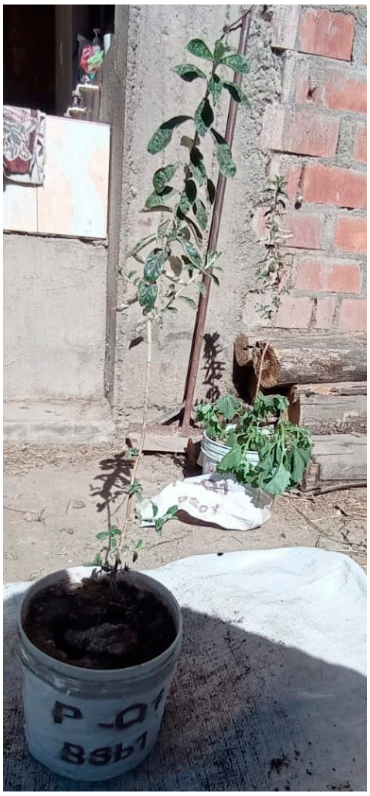

(c) 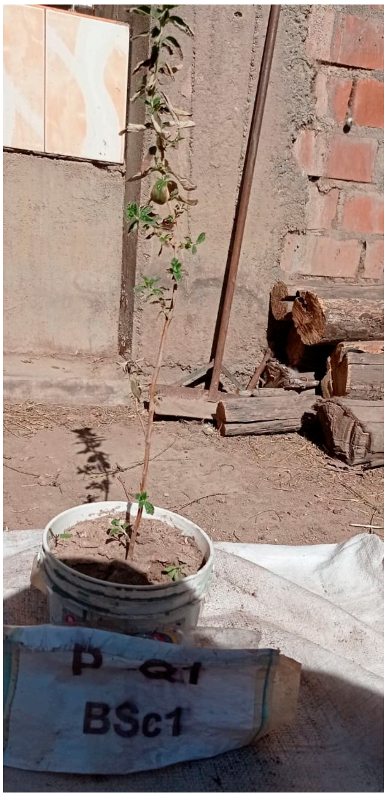

Figure S3. Photos of *Buddleja Species* L. in (a) pots with compost group "a" subgroup "1"; (b) pots with compost group "b" subgroup 1 and (c) pots with compost group "c" subgroup 1.

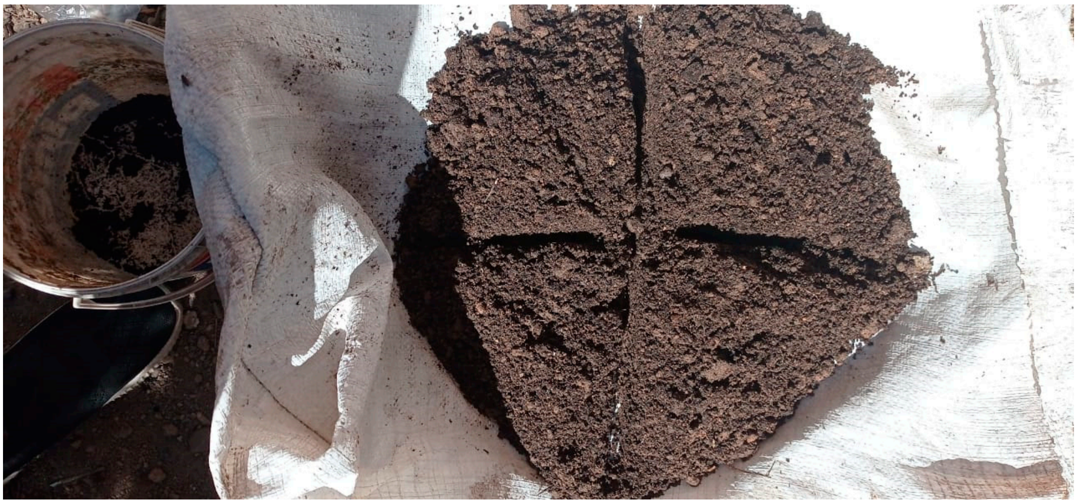

(a)

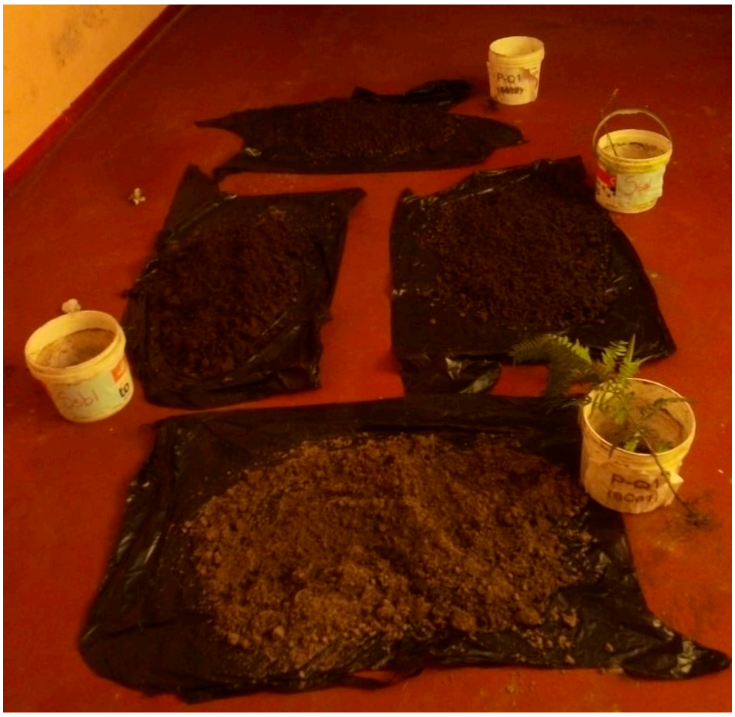

(b)

Figure S4. Photos of soil sampling of the first group for analysis at 60 days (a) soil and sampling of *Buddleja Species* pot and (b) soil and sampling of *Schinus Molle* L. pot.
